# Structure-Activity Relationships of a Series of Echinocandins and the Discovery of CD101, a Highly Stable and Soluble Echinocandin with Distinctive Pharmacokinetic Properties

**DOI:** 10.1128/AAC.01541-16

**Published:** 2017-01-24

**Authors:** Kenneth D. James, Christopher P. Laudeman, Navdeep B. Malkar, Radha Krishnan, Karen Polowy

**Affiliations:** Seachaid Pharmaceuticals, Inc., Durham, North Carolina, USA

**Keywords:** Candida, antifungal agents, antimicrobial agents, echinocandin, pharmacokinetics, pharmacology, structure-activity relationships

## Abstract

Echinocandins are a first-line therapy for candidemia and invasive candidiasis. They are generally safe with few drug interactions, but the stability and pharmacokinetic properties of currently approved echinocandins are such that each was developed for daily intravenous infusion. We sought to discover a novel echinocandin with properties that would enable more flexible dosing regimens, alternate routes of delivery, and expanded utility. Derivatives of known echinocandin scaffolds were generated, and an iterative process of design and screening led to the discovery of CD101, a novel echinocandin that has since demonstrated improved chemical stability and pharmacokinetics. Here, we report the structure-activity relationships (including preclinical efficacy and pharmacokinetic data) for the series of echinocandin analogs from which CD101 was selected. In a mouse model of disseminated candidiasis, the test compounds displayed clear dose responses and were generally associated with lower fungal burdens than that of anidulafungin. Single-dose pharmacokinetic studies in beagle dogs revealed a wide disparity in the half-lives and volumes of distribution, with one compound (now known as CD101) displaying a half-life that is nearly 5-fold longer than that of anidulafungin (53.1 h versus 11.6 h, respectively). *In vitro* activity data against panels of Candida spp. and Aspergillus spp. demonstrated that CD101 behaved similarly to approved echinocandins in terms of potency and spectrum of activity, suggesting that the improved efficacy observed *in vivo* for CD101 is a result of features beyond the antifungal potency inherent to the molecule. Factors that potentially contribute to the improved *in vivo* efficacy of CD101 are discussed.

## INTRODUCTION

Candida is the leading cause of bloodstream infections (BSIs) within U.S. hospitals and has been reported to account for 22% of inpatient BSIs (1). Candidemia, in particular, is associated with longer hospital stays, increased costs, and high morbidity and mortality. Overall mortality rates have been around 35% (2), but rates as high as 76% have been reported (3). The echinocandins are a first-line therapy against candidemia and some invasive Candida infections (4, 5). These agents act as noncompetitive inhibitors of the plasma membrane-bound β-1,3-d-glucan synthase enzyme complex, inhibiting the synthesis of a structural polymer (β-1,3-d-glucan) that comprises up to 60% of the cell wall of Candida spp. (6). The prevalence of this target in certain fungi, coupled with its absence in mammals, helps make the echinocandins very attractive in terms of low toxicity and reduced side effects. Also, the low incidence of resistance and virtually nonexistent drug interactions give the echinocandins advantages over other classes of antifungal drugs.

Despite these desirable features, the full therapeutic potential of echinocandins may yet be unrealized. Clearance rates *in vivo* and poor oral absorption of current echinocandins supported the approval of only once-daily administration by intravenous (i.v.) infusion, and poor stability has prevented the development of other dosage forms, such as topical or subcutaneous preparations. Consequently, the echinocandins presently receive little or no use for indications in which daily infusion of the drug is impractical.

**FIG 1:**
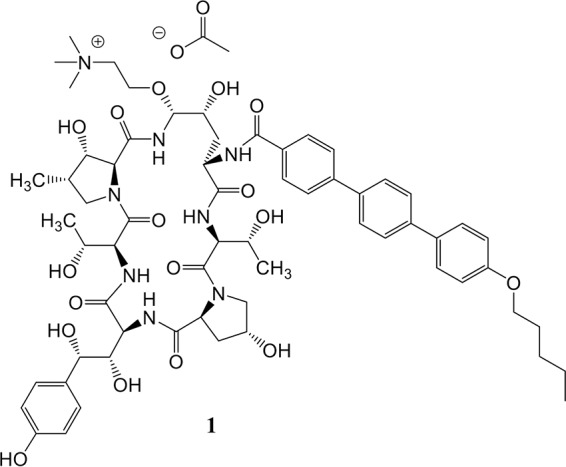
Structure of CD101 acetate.

**FIG 2:**
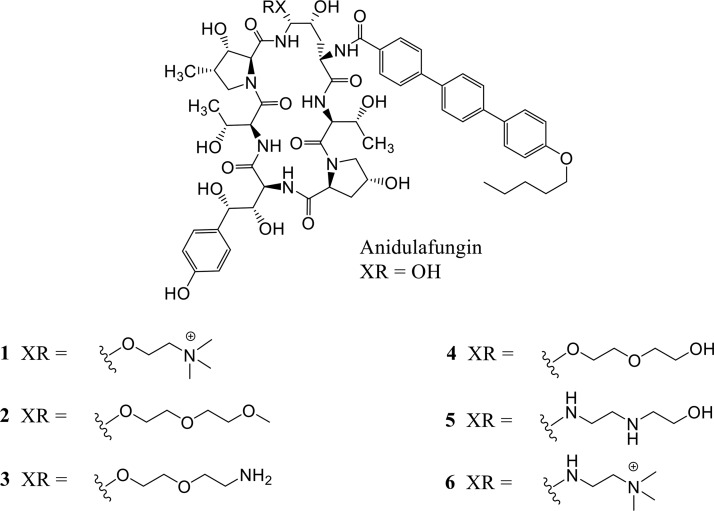
Structures of select echinocandin analogues and the derivatives described in these studies. Four of the compounds (compounds 1 to 4) are hemiaminal ethers; the other two (compounds 5 to 6) are aminals. Although they are structurally similar and nearly identical in molecular weight, the half-lives observed for these compounds varied greatly. Compound 1 (CD101) was selected for further characterization and development.

We sought to discover an echinocandin with properties that may allow for more flexible dosing regimens, alternate routes of delivery, and the expanded utility of this important drug class. An iterative process of rational design, synthesis, and screening resulted in the discovery of CD101 acetate (CD101; Fig. 1), a novel echinocandin with a potency and spectrum of activity *in vitro* typical of the echinocandins (7, 8) yet with a longer half-life (9, 10, 22; V. Ong, K. D. James, S. Smith, and B. R. Krishnan, submitted for publication) and an improved safety profile (11). CD101 is presently in phase 2 clinical development as a once-weekly i.v. formulation that provides high plasma exposure for treating candidemia (RADIANT trial [ClinicalTrials.gov identifier NCT02733432]) and as a topical formulation for treating acute and recurrent vulvovaginal candidiasis (STRIVE trial [ClinicalTrials.gov identifier NCT02734862]). Here, we report the structure-activity relationships of a select series of six echinocandin compounds, including CD101 (Fig. 2), and discuss the impact of similar moieties on activity and pharmacokinetics.

## RESULTS

### *In vivo* efficacy.

**FIG 3:**
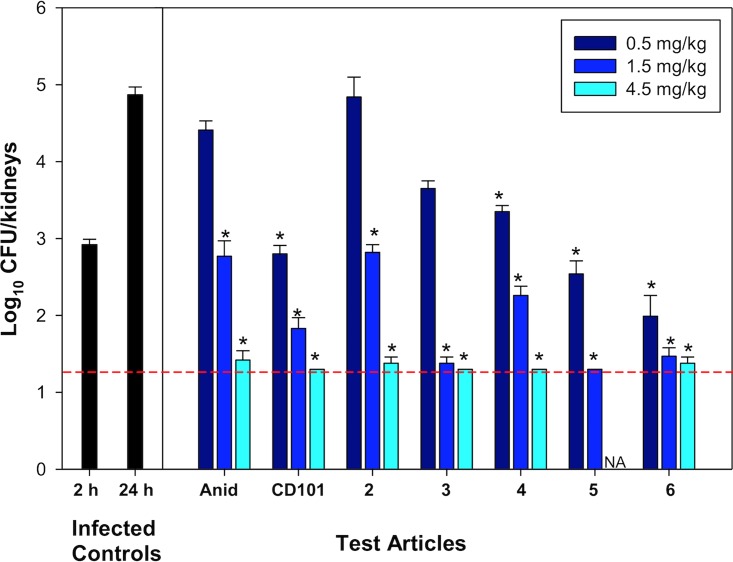
Fungal burdens in the kidneys of mice after infection with C. albicans. The black bars indicate fungal burden of infected, untreated controls at the time the treatment cohorts received test article (2 h) and at the time treatment cohorts were sacrificed (24 h after infection). All test articles displayed a good dose response. Fungal counts below the limit of detection (LOD) are reported at the LOD, indicated by the dashed red line. Anid, anidulafungin; NA, not available; *, *P* < 0.01.

The fungal burdens in the kidneys of CD-1 mice infected with Candida albicans and treated with various echinocandin test compounds are presented in Fig. 3. From 2 h to 24 h, the infected, untreated controls displayed a 2-log increase in fungal burden (CFU/kidneys). All treatment groups displayed a dose response in the assay. A dose of 1.5 mg/kg of body weight for each group resulted in at least a 2- to 3-log decrease in fungal burden over the untreated control at 24 h. All treatment cohorts receiving the 4.5-mg/kg dose had fungal burdens near or below the limit of detection. Four of the seven administered compounds (CD101, 4, 5, and 6) resulted in statistically significant (*P* < 0.01) reductions in fungal burden at all doses. Also, four of the seven administered compounds (CD101, 3, 4, and 5) resulted in fungal burdens below the limit of detection for at least one of the doses.

### Pharmacokinetics in dogs.

**FIG 4:**
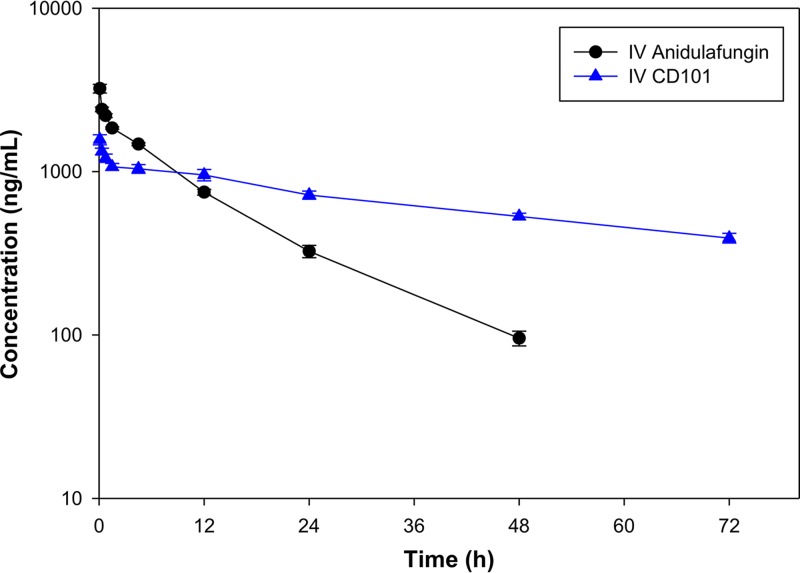
Plasma concentration-time curves for CD101 and anidulafungin in beagle dogs. Four nonnaive dogs received a single dose (1.4 mg/kg) of either CD101 (blue triangle) or anidulafungin (black circle) in a crossover fashion. Each point on the graph is an average from four animals plus or minus the standard error of the mean (SEM).

The half-lives (*t*_1/2_) and volumes of distribution (*V*) for anidulafungin, CD101, and other test compounds following i.v. administration (10-min slow bolus) in beagle dogs are presented in Table 1. The half-life for anidulafungin was 11.6 h. The half-lives for each of the test compounds were at least 50% longer than that for anidulafungin, with most of them being 2× to 3× longer. CD101 had the longest half-life (53.1 h), which was over 4.5-fold longer than that for anidulafungin. The volume of distribution for CD101 was the largest of all test articles at 1,360 ml/kg compared with 779 ml/kg for anidulafungin. The plasma concentration time curves for CD101 and anidulafungin are shown in Fig. 4. Clearance for CD101 was 19 ml/h/kg, whereas for anidulafungin it was 47 ml/h/kg.

**TABLE 1:** Half-lives and volumes of distribution for anidulafungin and select test articles after i.v. administration (10-min slow bolus) in beagle dogs

Test article	*t*_1/2_ (h)	*V* (ml/kg)
Anidulafungin	11.6	779
CD101	53.1	1,360
2	18.9	331
3	33.7	627
4	28.9	361
5	21	687
6	27.6	874

### *In vitro* activity.

The MIC values at 24 h and 48 h for CD101 and compound 6 against seven species of Candida are presented with data from approved comparator drugs in Table 2. These two compounds were specifically selected for this comparison because of their demonstrated *in vivo* efficacy, structural similarity, and disparate half-lives. CD101 and compound 6 were virtually indistinguishable in this assay, and both compounds tracked very well with anidulafungin in terms of potency and spectrum of activity. Values below 0.03 μg/ml were observed for the most common species. Values ranging from 0.25 to 2 μg/ml were observed for Candida lusitaniae, Candida guilliermondii, and Candida parapsilosis. As has been observed in other studies (7, 8), MIC values for caspofungin were generally slightly higher than those for the other echinocandins tested.

**TABLE 2:** MICs of CD101 and compound 6 with echinocandin and polyene comparators against Candida spp.

Organism	Time (h)	MIC (μg/ml)^*a*^				
		Anid	Caspo	CD101	6	AmpB
Candida albicans ATCC 90028	24	≤0.015	0.06	0.03	0.03	≤0.06
	48	≤0.015	0.06	0.03	0.03	0.125
Candida glabrata ATCC 90030	24	0.03	0.125	0.03	0.03	0.06
	48	0.03	0.125	0.03	0.03	0.25
Candida guilliermondii ATCC 34134	24	1	0.25	0.5	0.5	≤0.06
	48	2	>16^*b*^	2	1	≤0.06
Candida krusei ATCC 14243	24	0.03	0.25	0.03	0.03	0.25
	48	0.06	0.5	0.03	0.03	0.5
Candida lusitaniae ATCC 34134	24	0.125	0.25	0.25	0.25	≤0.06
	48	0.125	0.25	0.25	0.25	≤0.06
Candida parapsilosis ATCC 22019	24	0.5	0.25	0.5	0.25	0.25
	48	0.5	0.5	1	0.5	0.5
Candida tropicalis ATCC 90874	24	≤0.015	0.06	≤0.015	≤0.015	0.25
	48	≤0.015	0.06	≤0.015	≤0.015	0.25

a AmpB, amphotericin B; Anid, anidulafungin; Caspo, caspofungin.

b Reduced growth occurred, but the CLSI endpoint was not achieved.

The minimum effective concentrations (MECs) at 48 h for CD101, compound 6, and echinocandin comparators are presented with MICs of amphotericin B against seven species of Aspergillus in Table 3. As was the case with the testing against Candida, CD101 and compound 6 were indistinguishable from each other and from anidulafungin in terms of potency and spectrum of activity. MEC values for anidulafungin, CD101, and compound 6 were below 0.03 μg/ml against all Aspergillus species tested. MEC values were likewise higher for caspofungin than for the other echinocandins, but all were 0.125 μg/ml or lower.

**TABLE 3:** Minimum effective concentrations and MICs of CD101 and compound 6 with echinocandin and polyene comparators against Aspergillus spp.

Organism	Anid MEC (μg/ml)	Caspo MEC (μg/ml)	CD101 MEC (μg/ml)	Compound 6 MEC (μg/ml)	AmpB MIC (μg/ml)
Aspergillus candidus ATCC 13686	≤0.015	≤0.015	≤0.015	≤0.015	≤0.06
Aspergillus clavatus ATCC 10058^*a*^	≤0.015	0.125	0.03	0.03	≤0.06
Aspergillus flavus ATCC 22546	≤0.015	0.06	≤0.015	≤0.015	1
Aspergillus fumigatus ATCC 204305	≤0.015	0.125	≤0.015	≤0.015	1
Aspergillus niger ATCC 16888	≤0.015	0.06	≤0.015	≤0.015	0.125
Aspergillus ochraceus ATCC 96919	0.03	0.06	0.03	0.03	2
Aspergillus fumigatus ATCC MYA-3626	≤0.015	0.125	≤0.015	≤0.015	1

a Results for this organism were read at 68 h.

## DISCUSSION

Our objective was to discover an echinocandin with properties that may allow for more flexible dosing regimens, alternate routes of delivery, and expanded utility compared to the currently approved echinocandins. Such a development would potentially enable use in more widespread indications, including prophylaxis and vulvovaginal candidiasis. A suitable compound would need to retain the potency and spectrum of activity of the echinocandins while providing the pharmacokinetic properties and *in vivo* efficacy requisite for such usages.

Efficacy assays of the echinocandin test compounds in a murine candidiasis model demonstrated that some structural modifications at the C5 ornithine position were allowed without compromising efficacy compared to the unmodified anidulafungin (Fig. 3). This tolerance for structural modification was limited since many of the derivatives that were comprised of sterically bulky moieties demonstrated little or no activity (data not shown). The structural analogs and derivatives illustrated in Fig. 2 provide interesting structure-activity relationship information. The modifying moieties of these compounds are notably similar in size and molecular weight; however, they vary widely in the number of hydrogen bond acceptors and donors. Also of note, in this select group, two compounds have a permanent charge, two have a latent cationic charge at physiologic pH, and two are charge neutral. Yet, all of them performed at least as well as anidulafungin in this assay, and all demonstrated a good dose response. The compounds with aminals (including compounds 5 and 6) performed particularly well in the assay.

The performance of the compounds in this assay is a culmination of many factors, such as inherent anti-Candida potency, free fraction (based on protein binding), half-life (impacting overall exposure to the drug), distribution to the kidneys, and postantifungal effect (PAFE). The MIC and MEC data (Tables 2 and 3) suggest that the relative efficacies seen across these test compounds are not due to the greater inherent inhibition of the glucan synthase enzyme complex target. In contrast, the data of Table 1 and Fig. 4 suggest that increased half-lives and increases in exposure do contribute to increased efficacy *in vivo*. However, this assay alone does not shed light on the contributions of each of the aforementioned and other variables, such as maximum concentration of drug in plasma (*C*_max_) or area under the concentration-time curve (AUC), to the increase in efficacy *in vivo*. Additional work is being conducted to fully characterize the relationships between these variables and *in vivo* efficacy.

Anidulafungin was used as a comparator in the pharmacokinetic studies not only because of its structural similarity but also because it has the longest half-life among the approved echinocandins, both in clinical trials and in multiple animal models (12–16). The modifications at the C5 ornithine position all resulted in prolonged half-lives compared to that of anidulafungin, though the magnitudes of differences varied widely. A mechanistic explanation with respect to CD101 has already been proposed based on the unusual stability of the compound and the minimization of the chemical degradation pathway (17), which is a primary means of clearance for the echinocandins (18–21) and a source of reactive degradants that may contribute to toxicity (11). Nevertheless, examination of the data in Table 1 reveals the complexity of trying to predict half-lives. While the data suggest that the hemiaminal ethers and aminals result in a prolonged half-life compared to the hemiaminal of anidulafungin, the variation within and between these functional groups makes it less clear. The derivatives with the shortest and longest half-lives (compound 2 and CD101) were both hemiaminal ethers. CD101 features a quaternary amine, but compound 6 also has this group and, with respect to half-life, is moderate by comparison. Thus, neither the hemiaminal ether moiety nor the quaternary ammonium moiety alone explains the pharmacokinetic advantages of CD101.

The suitability of the dog model as a pharmacokinetic screen for these compounds has been supported by subsequent results in other species. The long half-life of CD101 relative to anidulafungin and other echinocandins has since been observed in the cynomolgus monkey (9) and chimpanzee (10). More importantly, this trend has been repeated in human studies. Compared to the half-life of 11.6 h in dogs, anidulafungin has shown a longer half-life in humans (24 to 26 h) (12, 13). Likewise, CD101 has recently demonstrated a longer half-life (>80 h) in clinical trials (22).

The modifications resulting in CD101 and compound 6 did not restrict the spectrum of activity against Candida spp. and Aspergillus spp. Likewise, the potencies against the tested organisms were comparable to those observed with anidulafungin, an observation that has since been further supported in studies against antifungal-resistant organisms (7, 23), resistance development studies (24), and surveillance studies (8). Thus, these modifications appeared to neither enhance nor diminish inherent *in vitro* activity. Mechanistic studies to further elucidate this are under way.

In conclusion, modifications of anidulafungin at the C5 ornithine position produced some echinocandin compounds that performed favorably compared to anidulafungin in a mouse model of disseminated candidiasis. The derivatizations that led to the most active compounds in this study all had approximately the same molecular weight but varied in hydrogen bond donors and acceptors and were comprised of moieties that were permanently charged, chargeable at physiologic pH, and charge neutral. The modifications producing each of the echinocandins reported in this study resulted in long half-lives compared to that of anidulafungin. In studies in beagle dogs, one member of this group, CD101, was distinct from the others in terms of an uncommonly long half-life and a comparatively large volume of distribution, both of which may prove beneficial to treating invasive fungal infections. Neither the hemiaminal ether moiety nor the quaternary ammonium moiety alone explains the pharmacokinetic advantages of CD101. Structurally similar compounds featuring only one of these moieties did not exhibit the same beneficial pharmacokinetic properties. These structure-activity relationships, together with the activity and pharmacokinetics of CD101, led to its selection for further investigation. CD101 is currently in development as a once-weekly i.v. treatment of candidemia and as the first echinocandin candidate for topical treatment of vulvovaginal candidiasis. The discovery of CD101 may expand the use and utility of echinocandins in the treatment of invasive fungal infections and for diseases not previously considered for echinocandin therapy.

## MATERIALS AND METHODS

### Test articles.

Comparator compounds were obtained from commercial sources. All six test compounds were synthesized and purified as previously described (25). The purity of each synthesized compound was determined by reversed-phase high-performance liquid chromatography (HPLC). An Agilent Pursuit XRs diphenyl column (4.6-mm inside diameter [i.d.] by 250-mm length, 3.0-μm particle size) was used. Elution was performed with a column temperature of 40°C and a gradient system with a flow rate of 1 ml/min (eluent A, H_2_O with 0.1% trifluoroacetic acid [TFA]; eluent B, acetonitrile with 0.1% TFA; %B: 10% to 42% over 5 min, 42% to 50% over 20 min). UV detection was performed at 300 nm. Test compounds and comparators were used uncorrected for moisture content.

### *In vivo* efficacy.

Candida albicans R303 was grown on Sabouraud dextrose agar (SDA) plates for 24 h at 35°C. Colonies were transferred to phosphate-buffered saline (PBS) and diluted to 3.8 × 10^5^ CFU/ml. Female CD-1 mice (Charles River Laboratories, Portage, MI) were rendered neutropenic via injection with cyclophosphamide (150 mg/kg) 5 days and 2 days prior to inoculation. Each animal was infected by injecting 0.1 ml of inoculum into the tail vein. In the treatment groups, animals received 0.5, 1.5, or 4.5 mg/kg of test article 2 h after inoculation. Two groups of untreated, infected controls were used to evaluate fungal burden: at 2 h when test articles were administered in the treatment groups and at 24 h when the treatment groups were sacrificed. Kidneys were harvested at 2 h in one untreated control group and at 24 h in all other groups. The kidneys were homogenized in 2 ml of sterile phosphate-buffered saline, and 0.1-ml aliquots were spread on SDA plates and incubated at 35°C overnight. Fungal burden was determined based on the log_10_ C. albicans CFU counts from paired kidneys. Data were analyzed using a one-way analysis of variance (ANOVA) and the Dunnett multiple comparisons test comparing the test articles to the infected control group (GraphPad InStat version 3.1; GraphPad Software, San Diego, CA).

### Pharmacokinetics in dogs.

Four male, nonnaive beagle dogs received a single i.v. infusion (1.4 mg/kg) of test article over 10 min via a catheter placed in the cephalic vein. Blood draws (1.0 ml) were taken at 0.083, 0.33, 0.75, 1.5, 4.5, 12, 24, and 48 h. An additional blood draw at 72 h was obtained for CD101. Blood draws were taken from the jugular vein and were collected into K_3_ EDTA tubes. Following blood collection, the samples were immediately inverted several times and were held on wet ice pending centrifugation. The samples were centrifuged within ∼30 min of collection under refrigeration (∼5°C for ∼10 min at ∼2,000 g) to obtain plasma. The plasma samples were frozen immediately on dry ice after separation and were stored at approximately −70°C until analysis. Levels of test article in the plasma samples were measured by quantitative liquid chromatography-tandem mass spectrometry (LC-MS/MS) analysis compared to a calibration curve and an internal standard. Pharmacokinetic parameters were calculated from the plasma concentration-time data using standard noncompartmental methods and utilizing WinNonlin analysis software.

### Animal care.

Mice and dogs used in these studies were housed and handled in accordance with the *Guide for the Care and Use of Laboratory Animals* (26). Protocols were approved by an Institutional Animal Care and Use Committee before experiments were performed. The number of animals was kept to the minimum considered necessary to differentiate between results of the test articles. Administration of test articles were kept at levels anticipated to be therapeutically relevant at the time of the study.

### *In vitro* activity.

All yeast and mold isolates were obtained from the American Type Culture Collection (ATCC). Isolates were plated on an agar medium to verify purity, and single colonies were picked. Aspergillus isolates were resubcultured on potato dextrose slants and incubated for 7 days at 35°C. Candida isolates were streaked on SDA plates and incubated for 24 h at 35°C. RPMI 1640 medium (HyClone Laboratories, Logan, UT) and the inoculum for each organism were prepared according to Clinical and Laboratory Standards Institute (CLSI) guidelines (27–29). Stock solutions for all test articles were prepared in 100% dimethyl sulfoxide (DMSO). Susceptibility testing was performed using CLSI broth microdilution methods for Candida (M27-A3) and Aspergillus (M38-A2). Amphotericin B was tested at a range of 0.06 to 64 μg/ml; all other antifungal agents were tested at 0.015 to 16 μg/ml. Plates were incubated at 35°C and were read from the bottom using a plate viewer after 24 and 48 h for Candida and after 48 h for Aspergillus. MIC values are reported as the lowest drug concentration to yield a 50% reduction in the growth of Candida species tested compared to the control well. Minimum effective concentration (MEC) values are reported as the lowest drug concentration to yield the stunted hyphal growth of Aspergillus species tested compared to that of the control well.
